# A 5-Genomic Mutation Signature Can Predict the Survival for Patients With NSCLC Receiving Atezolizumab

**DOI:** 10.3389/fimmu.2021.606027

**Published:** 2021-06-23

**Authors:** Jiamao Lin, Xiaohui Wang, Chenyue Zhang, Shuai Bu, Chenglong Zhao, Haiyong Wang

**Affiliations:** ^1^ Department of Internal Medicine Oncology, Shandong Cancer Hospital and Institute, Shandong First Medical University and Shandong Academy of Medical Sciences, Jinan, China; ^2^ Research Service Office, Shandong Liaocheng People’s Hospital, Liaocheng, China; ^3^ Department of Integrated Oncology, Fudan University Shanghai Cancer Center, Shanghai, China; ^4^ Research Department, The Second Affiliated Hospital of Shandong University of Traditional Chinese Medicine, Jinan, China; ^5^ Department of Pathology, Shandong Cancer Hospital and Institute, Shandong First Medical University and Shandong Academy of Medical Sciences, Jinan, China

**Keywords:** atezolizumab, gene mutation, non-small cell lung cancer, survival, PD-L1 inhibitor

## Abstract

**Background:**

At present, there is a lack of studies focusing on the survival prediction of patients with non-small cell lung cancer (NSCLC) receiving atezolizumab in light of gene mutation characteristic.

**Methods:**

Patients with NSCLC receiving atezolizumab from the OAK study were defined as the training group. LASSO Cox regressions were applied to establish the gene mutation signature model to predict the overall survival (OS) rate of the training group. NSCLC patients receiving atezolizumab from the POPLAR study were defined as the testing group to validate the gene mutation signature model. In addition, we compared the OS rate between patients receiving atezolizumab and docetaxel classified according to their risk score based on our gene mutation signature model.

**Results:**

We successfully established a 5-genomic mutation signature that included *CREBBP*, *KEAP1*, *RAF1*, *STK11* and *TP53* mutations. We found it was superior to the blood tumor mutation burden (bTMB) score and programmed death ligand 1 (PDL1) expression in the prediction of the OS rate for patients receiving atezolizumab. High-risk patients receiving atezolizumab had a worse OS rate compared with low-risk patients in the training (*P* = 0.0004) and testing (*P* = 0.0001) groups. In addition, low-risk patients using atezolizumab had a better OS rate compared with those in use of docetaxel for the training (*P <*0.0001) and testing groups (*P* = 0.0095). High-risk patients of the training group (*P* = 0.0265) using atezolizumab had a better OS rate compared with those using docetaxel. However, the OS difference between atezolizumab and docetaxel was not found in high-risk patients from the testing group (*P* = 0.6403). Multivariate Cox regression analysis showed that the risk model in light of 5-genomic mutation signature was an independent prognostic factor on OS for patients receiving atezolizumab (*P <*0.0001). In addition, significant OS benefit could only be found in low-risk patients receiving atezolizumab compared with docetaxel (*P <*0.0001).

**Conclusions:**

The 5-genomic mutation signature could predict OS benefit for patients with NSCLC receiving atezolizumab. Therefore, the establishment of the 5-genomic mutation panel will guide clinicians to identify optimal patients who could benefit from atezolizumab treatment.

## Introduction

Atezolizumab, an immune checkpoint inhibitor, is an immunoglobulin G1 monoclonal antibody that binds to programmed death ligand 1 (PD-L1) and blocks its interactions with programmed death 1 and B7.1 receptor (1). At present, atezolizumab plays an important role in the treatment of non-small cell lung cancer (NSCLC) patients, gradually shifting from the second to the first line of treatment. Importantly, the tumor mutational burden (TMB) score and PDL1 expression have become key markers of the clinical benefits of patients receiving atezolizumab. Indeed, TMB in blood (bTMB) was identified as a biomarker for patients that improve upon atezolizumab treatment (2). The POPLAR study also found that improvements in the survival rates were associated with PDL1 expression on tumor cells and tumor-infiltrating immune cells, suggesting that PDL1 expression is can predict benefits derived from atezolizumab treatment (3). However, it is worth noting that the benefits of atezolizumab are comparable with those of chemotherapy based on these markers. In fact, increased overall survival (OS) rates were not obtained for patients receiving atezolizumab with a high bTMB score compared with those with low bTMB score from both the OAK and POPLAR studies (2, 4). A recent study even showed that advanced NSCLC patients with low TMB might have better OS than those with medium TMB (5). Therefore, it is very important to identify markers for the prediction of patients with NSCLC that could benefit from atezolizumab treatment.

Several studies have focused on the relationship between specific gene mutations and the effect of immunotherapy. A series of gene mutations such as *STK11*, *KEAP1*, *POLD1/POLE*, and *TERT* were found to influence the outcomes of patients receiving immunotherapy (6–10). Thus, we speculated that the establishment of a specific gene mutation signature could distinguish survival differences for patients receiving atezolizumab. In our study, we first established the gene mutation signature model able to predict OS rate based on the OAK study. Then, the gene mutation signature model was validated using data from the POPLAR study. Our study would be beneficial to guide the treatment of NSCLC patients receiving atezolizumab. It would also render the treatment strategy more individual-oriented.

## Methods

### Patient Data

The data of our study was obtained from a previous study (2). Our study was based on POPLAR and OAK studies, the two independent clinical trials. The POPLAR study is a multicentre, open-label, phase 2 randomized controlled trial to compare atezolizumab with docetaxel for patients with previously treated NSCLC (3). The OAK study is the first randomized phase 3 study reporting results of atezolizumab treatment, which resulted in a clinically relevant improvement of OS versus docetaxel in previously treated NSCLC (11). These relevant studies and data have been published, thus informed consent and ethical committee approval were not warranted.

### Study Design

**Figure 1:**
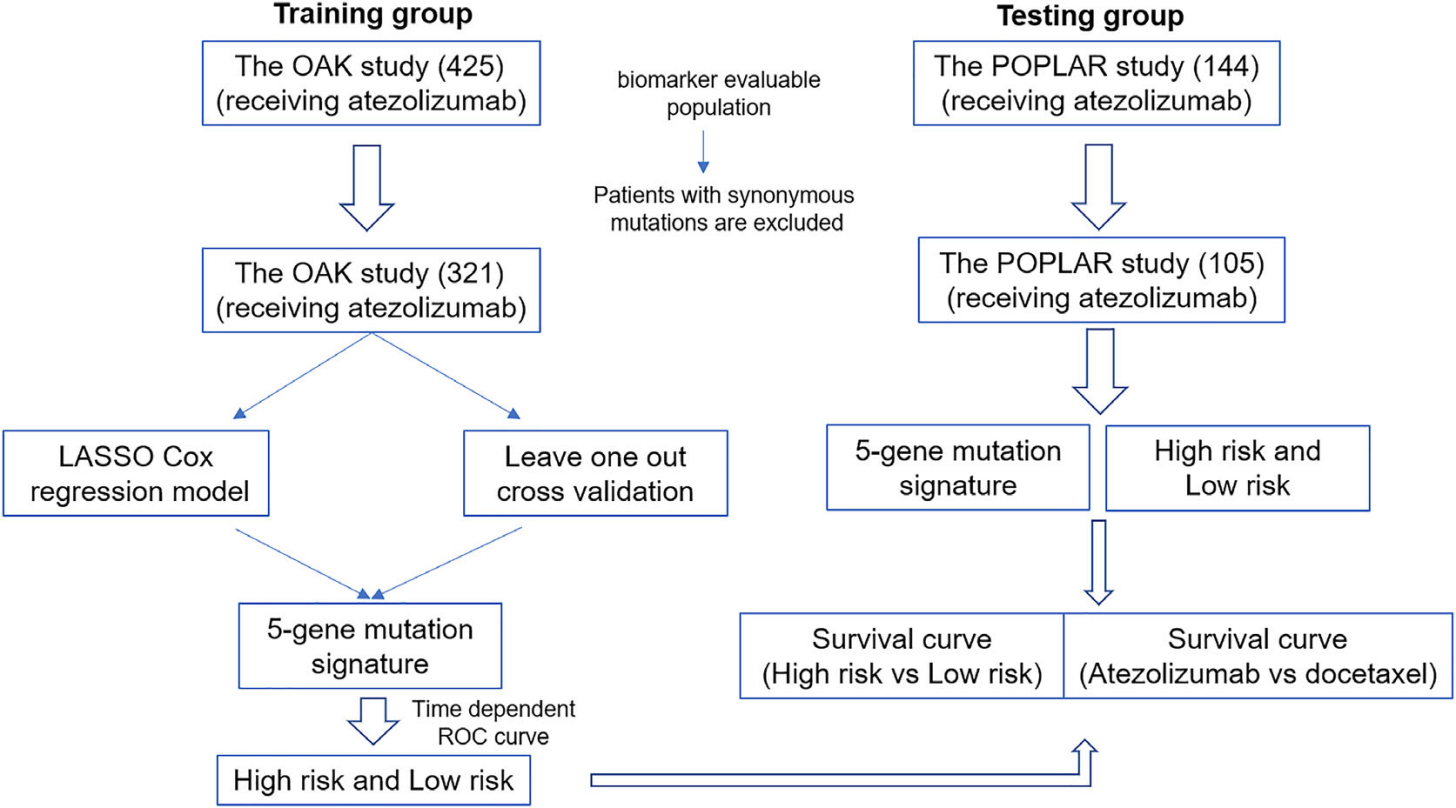
Research and design flow chart.

This study was divided into the training group and the testing group as shown in **Figure 1**. Patients with synonymous mutations, which are that sometimes a mutation of a base pair in a DNA fragment does not change the encoded amino acid, were excluded. The synonymous mutations have been defined in published data (2). A total of 321 patients receiving atezolizumab were included from the training group (OAK study) and 105 patients were included from the testing group (POPLAR study). LASSO Cox regression model was used to predict prognosis-related markers from the training group. Then leave one out cross validation was applied to select five optimal gene mutation types to construct a risk sore evaluation model. The risk score was calculated according to the formula: Σ_i_ *ω_i_ χ_i_* where *ω_i_* is the coefficient and *χ_i_* is the expression value of each respective gene. The optimal cutoff to distinct high- and low-risk was estimated through time-dependent ROC curves. OS was regarded as the main endpoint of our study.

### Statistical Analysis

The Kaplan–Meier curve was constructed to compare the OS difference, and the log-rank was used to perform the statistical analysis. Multivariate Cox regression analysis was applied to develop the subgroup analysis based on clinical variables. The construction of the risk score model was based on R 3.4.2. The figures were drawn using GraphPad Prism version 6.0. All *P*-values were two-tailed, and *P <*0.05 was considered statistically significant.

## Results

### Construction of a Genetic Mutation Signature to Predict the Survival of Patients Receiving Atezolizumab

**Figure 2:**
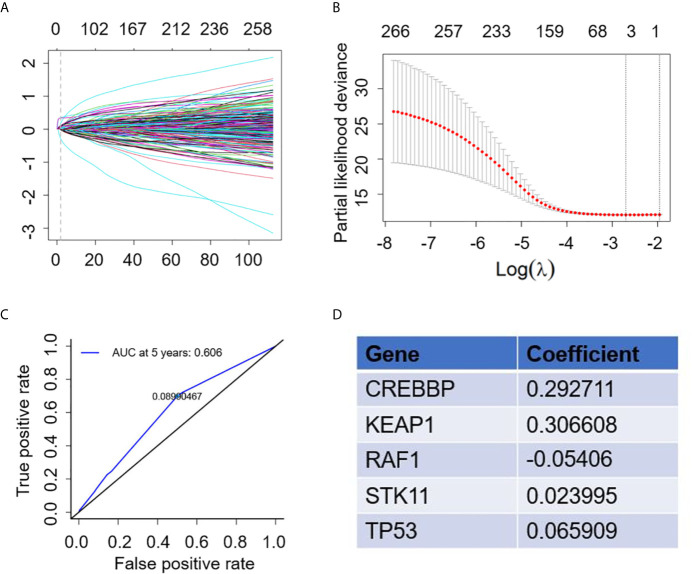
Construction of gene mutation signature to predict survival. **(A)** Trend graph of LASSO coefficients. **(B)** Partial likelihood deviation map. **(C)** Time dependent ROC curve. **(D)** 5 gene mutation types and matched coefficient.

A total of 321 NSCLC patients receiving atezolizumab from the OAK study were included in the analysis to establish the gene mutation signature. To achieve better stability and accuracy, we constructed the trend diagram of the lasso coefficient, and found a 5-genomic mutation signature obtained from 10 cross validations that could predict survival (**Figures 2A, B**). In addition, the time-dependent receiver operating characteristic (ROC) curve was applied to confirm the optimal cutoff value (0.08990467) to divide patients in high- and low-risk (**Figure 2C**). Based on the above screening, the five optimal gene mutations were: *CREBBP*, *KEAP1*, *RAF1*, *STK11* and *TP53* (**Figure 2D**). The detailed risk score is presented in **Supplementary Material 1**.

### Difference in bTMB Score and PDL1 Expression High- and Low-Risk Patients Based on Our Prediction Model

**Figure 3:**
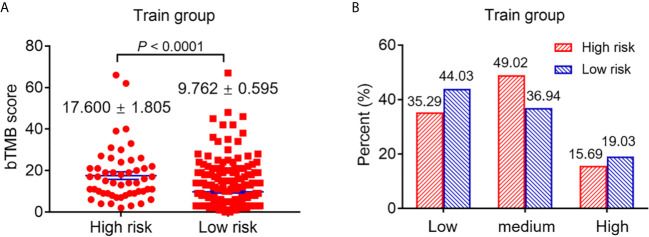
The difference of bTMB score and PDL1 expression of patients with high and low risk. **(A)** The difference of bTMB score of patients with high and low risk (*P < *0.0001). **(B)** The difference of PDL1 expression of patients with high and low risk.

Markers such as bTMB and PDL1 are vital to predict the efficacy of atezolizumab. In our study, we found that high-risk patients in the training group had a higher bTMB score compared with low-risk (high-risk 17.600 ± 1.805 *vs* low-risk 9.762 ± 0.595; *P <*0.0001) (**Figure 3A**). In addition, the PDL1 expression was divided into low (tumor cell (TC) <1%/immune cell (IC) <1%), medium (TC = 1–50%%/IC = 1–5%) and high (TC ≥50%/IC ≥5%) according to a previous study (3). The results showed that the PDL1 expression of high-risk patients was low in 35.29%, medium in 49.02% and high in 15.69% of patients, while for low-risk patients the PDL1 expression was low in 44.03%, medium in 36.94% and high in 19.03% of patients (**Figure 3B**).

### The Survival Analysis for the Training Group Based on the Gene Mutation Signature

**Figure 4:**
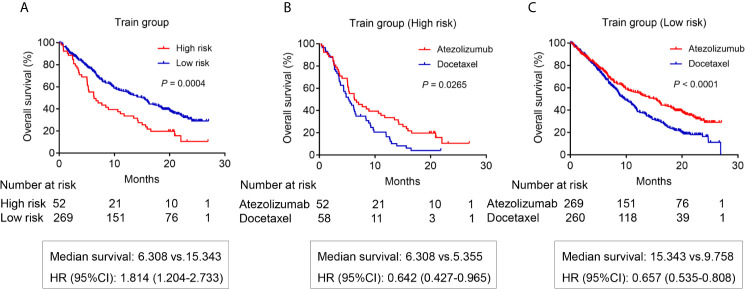
The OS analysis for patients in the training group from OAK study. **(A)** The OS difference between the high risk and low risk patients receiving atezolizumab (*P* = 0.0004). **(B)** The OS difference of high-risk patients between atezolizumab and docetaxel (*P* = 0.0265). **(C)** The OS difference of low-risk patients between atezolizumab and docetaxel (*P <* 0.0001).

The survival analysis was used to compare the OS rate of high- and low-risk patients receiving atezolizumab. The results showed that low-risk patients had a better OS rate with a median survival of 15.343 months compared with high-risk patients that had a median survival of 6.308 months (*P* = 0.0004) (**Figure 4A**). In fact, the OS benefit of low-risk patients was demonstrated in patients receiving docetaxel from the OAK study (*P <*0.0001) (**Supplementary Figure 1A** and **Supplementary Material 2**). Importantly, bTMB was not a predictor of OS of patients receiving atezolizumab (*P* = 0.647) (**Supplementary Figure 2A**). Patients receiving atezolizumab with PDL1 ≥1% also did not present differences in OS compared with those with PDL1 <1% (*P* = 0.479) (**Supplementary Figure 2B**). Patients receiving atezolizumab with PDL1 ≥50% showed a 10.5 months median OS benefit compared with those with PDL1 <50% (*P* = 0.0058) (**Supplementary Figure 2C**). Importantly, compared with PDL1 and bTMB, our risk model had a higher Hazard Ratio (HR) and C-index to predict the OS of patients receiving atezolizumab (**Supplementary Table 1**).

Next, we analyzed the OS difference for patients receiving atezolizumab compared with those subjected to docetaxel. The results showed the high-risk patients receiving atezolizumab had a better OS benefit with a median survival of 6.308 months compared with patients receiving docetaxel that presented a 5.355 months survival (*P* = 0.0265) (**Figure 4B**). Importantly, the OS benefit of atezolizumab *vs* docetaxel was more obvious for low-risk patients (*P <*0.0001) (**Figure 4C**).

### The Survival Analysis for Testing Group Patients Based on the Gene Mutation Signature

**Figure 5:**
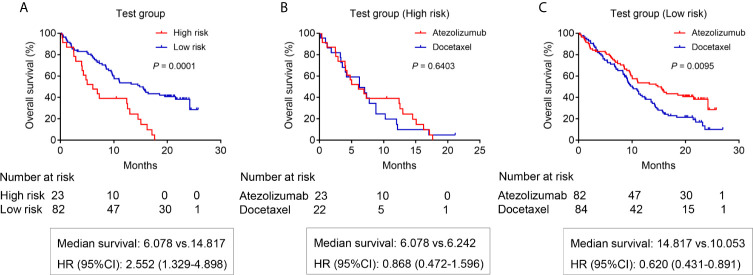
The OS analysis for patients in the testing group from POPLAR study. **(A)** The OS difference between the high risk and low risk patients receiving atezolizumab (*P* = 0.0001). **(B)** The OS difference of high-risk patients between atezolizumab and docetaxel (*P* = 0.6403). **(C)** The OS difference of low-risk patients between atezolizumab and docetaxel (*P* = 0.0095).

We attempted to further verify the feasibility of our prediction model using patients from the POPLAR study as our testing group. First, we screened patients receiving atezolizumab based on the same criteria (**Supplementary Material 3**). As expected, the results showed that low-risk patients receiving atezolizumab had a better OS (14.817 months) compared with high-risk patients (OS 6.078 months) (*P* = 0.0001) (**Figure 5A**). We also screened the patients receiving docetaxel from the POPLAR study (**Supplementary Material 4**). High-risk patients receiving docetaxel showed no OS benefits when compared to those in use of atezolizumab (median survival: 6.078 months *vs* 6.242 months, respectively) (*P* = 0.6403) (**Figure 5B**). However, low-risk patients receiving atezolizumab showed a better OS benefit with a median survival of 14.817 months compared with low-risk patients receiving docetaxel (median survival of 10.053 months) (*P* = 0.0095) (**Figure 5C**). In addition, the OS benefit of low-risk patients was also demonstrated in patients receiving docetaxel (*P* = 0.0063) (**Supplementary Figure 1B**).

### Subgroup Analysis on OS After Adjusting Clinical Variables for Patients Receiving Atezolizumab Based on Training and Testing Group

Importantly, we conducted multivariate Cox analysis by adjusting clinical variables including age, race, sex, ECOG, Histopathology and smoking history based on the training and testing group. The results showed that risk model based on the 5-genomic mutation signature was an independent prognostic factor for patients receiving atezolizumab (HR: 95%CI: 2.031 (1.528–2.700; *P <*0.0001) (**Table 1**). Likewise, we also analyzed the prognostic factors for patients receiving docetaxel, the result also demonstrated that risk model based on the 5-genomic mutation signature was also an independent prognostic factor for these patients (HR:95%CI: 1.995 (1.528–2.604; *P <*0.0001) (**Supplementary Table 2**). Importantly, the results of multivariate Cox regression analysis showed that atezolizumab had better OS benefit compared with docetaxel in the low-risk patients (HR:95%CI: 1.572 (1.312–1.883; *P <*0.0001) (**Table 2**). However, the positive connection between treatment and OS was not found in high-risk patients in light of same analysis (HR:95%CI: 1.387 (0.980–1.962; *P* = 0.065) (**Table 3**). Interestingly, we found there is an obvious interaction between smoking history and treatment in low-risk patients based on training and testing group (*P* = 0.024) (**Table 2**).

**Table 1:** Univariate and multivariate Cox regression analysis of clinical variables affecting OS for patients receiving atezolizumab based on training and testing group.

Variables	Univariate analysis		Multivariate analysis		Interaction*
	Wald	*P*	HR (95% CI)	*P*	*P*
Age	0.005	0.944	NI		0.692
<65				
≥65			
Race	1.656	0.198	NI		0.107
White					
Asian					
Others					
Sex	3.163	0.075	NI		0.496
Female					
Male					
Histopathology	7.668	0.006		0.005	0.806
Squamous			Reference		
Non-squamous			0.694 (0.537–0.896)	0.005	
ECOG	17.591	<0.0001		<0.0001	0.242
0			Reference		
1			1.757 (1.353–2.281)	<0.0001	
Smoking	4.242	0.039		0.180	0.098
Current			Reference		
Never			1.011 (0.649–1.576)		
Previous			1.285 (0.918–1.799)		
Risk model	25.125	<0.0001		<0.0001	
Low risk			Reference		
High risk			2.031 (1.528–2.700)	<0.0001	

NI, not included; *Interaction between variables and risk model.

**Table 2:** Univariate and multivariate Cox regression analysis of clinical variables affecting OS for low-risk patients based on training and testing group.

Variables	Univariate analysis		Multivariate analysis		Interaction*
	Wald	*P*	HR (95% CI)	*P*	*P*
Age	1.991	0.158	NI		0.198
<65					
≥65					
Race	2.269	0.132	NI		0.359
White					
Asian					
Others					
Sex	2.270	0.132	NI		0.826
Female					
Male					
Histopathology	15.777	<0.0001		<0.0001	0.720
Squamous			Reference		
Non-squamous			0.697 (0.577–0.842)	<0.0001	
ECOG	35.196	<0.0001		<0.0001	0.693
0			Reference		
1			1.833 (1.503–2.235)	<0.0001	
Smoking	3.367	0.067	NI		0.024
Current					
Never					
Previous					
Treatment	22.943	<0.0001		<0.0001	
Atezolizumab			Reference		
Docetaxel			1.572 (1.312–1.883)	<0.0001

NI, not included; *Interaction between variables and risk model.

**Table 3:** Univariate and multivariate Cox regression analysis of clinical variables affecting OS for high-risk patients based on training and testing group.

Variables	Univariate analysis		Multivariate analysis		Interaction*
	Wald	*P*	HR (95% CI)	*P*	*P*
Age	0.339	0.560	NI		0.800
<65					
≥65					
Race	2.066	0.151^#^		0.326	0.063
White			Reference		
Asian			0.663 (0.384–1.146)	0.141	
Others			0.868 (0.435–1.731)	0.687	
Sex	0.623	0.430	NI		0.214
Female					
Male					
Histopathology	1.540	0.215	NI		0.542
Squamous					
Non-squamous					
ECOG	1.699	0.192^#^		0.207	0.673
0			Reference		
1			1.270 (0.876–1.839)	0.207	
Smoking	0.297	0.586	NI		0.542
Current					
Never					
Previous					
Treatment	4.676	0.031		0.065	
Atezolizumab			Reference		
Docetaxel			1.387 (0.980–1.962)	0.065	

NI, not included; *Interaction between variables and treatment; ^#^P < 0.2 was included in multivariate analysis.

## Discussion

The efficacy rate of immunotherapy is relatively low, which has made it imperative to explore markers to predict the efficacy of immunotherapy. It has been commonly acknowledged that high TMB and PD-L1 is associated with improved prognosis. However, there have been some limitations in their prediction of immunotherapy responses. Recently, a growing number of studies have focused on the role of genetic mutations in the prediction of immunotherapy. To explore the impact of genetic mutation on ICI prediction, we therefore developed a risk score model based on gene mutations to predict the OS of patients receiving atezolizumab. The clinical significance of our findings could be seen in three aspects: First, our 5-genomic mutation signature was demonstrated to be a better predictor than PDL1 and bTMB to screen patients who would benefit from atezolizumab; It is also very convenient and economical to detect; More importantly, our 5-genomic mutation signature was able to predict OS rates of patients receiving atezolizumab, which may help clinicians select patients who could benefit more from such therapy.

The 5-genomic mutation signature of our risk score model consisted of mutations in *CREBBP*, *KEAP1*, *RAF1*, *STK11* and *TP53*. *CREBBP*, which encodes an acetyltransferase, has been frequently found to develop mutations in many tumor types (12–14). At present, there is no literature focusing on the relationship between the *CREBBP* mutation and immunotherapy outcomes. Only one previous study showed that loss of function of *CREBBP* resulted in focal depletion of enhancer *H3K27* acetylation and aberrant transcriptional silencing of genes that regulate B-cell signaling and immune responses, such as class II MHC (15). *HDAC3* inhibition represents a novel mechanism-based immune epigenetic therapy for lymphomas caused by *CREBBP* mutation (16). *KEAP1* is located at 19p13.2, and its protein has three major domains: an N-terminal broad complex, tram track, and the bric-a-brac (BTB) domain; a central intervening region (IVR); and a series of six C-terminal Kelch repeats (17). High-frequency mutations in *KEAP1* have been identified in Chinese patients with lung squamous cell carcinoma, while the somatic nonsynonymous mutation of *KEAP1* in patients with lung cancer is likely to promote tumorigenesis *via* activation of the *KEAP1/NRF2* antioxidant stress response pathway (18). Our previously study revealed that *KEAP1*-mutant NSCLC is associated with higher TMB, and also found that the OS was prolonged in NSCLC patients receiving immunotherapy with wild-type *KEAP1* compared with a mutant (6). Another study has demonstrated that *STK11/KEAP1* mutations may help identify bTMB-high patients unlikely to respond to pembrolizumab (19). Indeed, *STK11/KEAP1* mutations are prognostic, not predictive, biomarkers for anti-PD-1/anti-PDL1 therapy (20). Interestingly, we previously analyzed the relationship between *STK11* mutation and immune-related prognostic markers and immune microenvironment. The results showed that patients with the *STK11* mutation did not benefit from immune checkpoint inhibitors (6).

At present, there are a few studies focusing on *RAF1* mutation and no literature reporting the relationship between *RAF1* mutation and immunotherapy. In parallel, *BRAF* mutation was found in lung cancer even though its association with immunotherapy efficacy is controversial (21–23). *TP53* mutation is common in patients with lung cancer. Many studies focused on the relationship between *TP53* mutation and immunotherapy efficacy. Assoun et al. reported that *TP53* mutation was associated with OS benefits in NSCLC patients with advanced non-small cell lung cancer treated with immune checkpoint inhibitor (24). A study from China also demonstrated that *TP53* and *KRAS* mutations in lung adenocarcinoma might serve as a pair of potential predictive factors to guide anti-PD-1/PDL1 immunotherapy (25). Altogether, the relationship between *TP53* and immunotherapy still needs further investigation.

We have successfully established a risk model based on the five genomic mutations exposed above. And the risk of death is significantly higher in high-risk cohort compared with low-risk for those receiving atezolizumab. High-risk patients were found to be those with high bTMB and PD-L1, indicating bTMB and PD-L1 were not accurate to distinguish OS for patients receiving atezolizumab. One study led by Nie also demonstrated that advanced NSCLC patients with low tumor mutation burden might derive benefit from immunotherapy (5), as consistent with our study. In fact, we compared the HR of different OS prediction models and found that our 5-genomic mutation signature had a higher HR in both the POPLAR and OAK studies when compared to other prediction models. A higher C-index was also found for our model of 5-genomic mutation signature. These results suggest that our risk score model is a better predictor of OS rate when compared to bTMB and PDL1 expression. However, more data are needed to verify our conclusions.

Another interesting finding is that our risk model could also predict the OS of patients receiving docetaxel. This may be due to the fact that these specific gene mutations are involved in the malignant biological transformation of tumors, which may not be sensitive to treatment. High-risk patients receiving atezolizumab showed a better OS compared with those treated with docetaxel from the OAK study, however, the OS benefit was not found in the POPLAR study. Maybe this is due to the smaller sample size of the POPLAR study. Moreover, there was only a one month OS benefit for high-risk patients receiving atezolizumab compared with those using docetaxel within the OAK study, which may indicate that high-risk patients have limited benefits from atezolizumab. The follow-up multivariate Cox regression analysis further proves our conjecture when combining OAK and POPLAR studies after adjusting clinical variables.

Undeniably, it has to be noted that there are some limitations of our study that need to be addressed. First, all the gene mutation types were obtained by liquid biopsies in our study, which means some gene mutations were lost. If the 5-genomic mutation signature based on liquid biopsies could be successfully verified in the future, then it could have wider clinical applications. Second, the 5-genomic mutation signature based on the OAK and POPLAR cohorts was established only to predict OS benefit for NSCLC patients receiving atezolizumab. Due to the uniqueness of PD1/PDL1 inhibitors, it is necessary to further study whether our risk score model is suitable for other immune checkpoint inhibitors. Last but not the least, we performed a data analysis based on the published OAK and POPLAR cohorts and further verification is needed in the future. And there have been several studies focusing on the role of nutritional and inflammatory indexes in the prediction of survival for patients receiving atezolizumab (26, 27). Our study distinguished from them by centering on genetic mutations. These studies are not contradictory but rather complementary to each other. Despite these limitations, our prediction model for atezolizumab still holds clinical relevance for its superiority in ICI prediction than TMB and PD-L1, as confirmed in the present study.

In conclusion, we successfully established a 5-genomic mutation signature risk score model to predict the OS rate of patients receiving atezolizumab. Importantly, low-risk patients were more likely to benefit from atezolizumab compared with those treated with docetaxel. It would be beneficial to develop a gene mutation panel to guide the treatment of NSCLC patients receiving atezolizumab in the future.

## Data Availability Statement

The original contributions presented in the study are included in the article/**Supplementary Material**. Further inquiries can be directed to the corresponding author.

## Author Contributions

Study design: HW. Date collection: JL and XW. Date analysis: CyZ. Writing: JL, SB and ClZ. Chart making: JL, XW, CyZ, SB and ClZ. Supervision and amendment: HW. All authors contributed to the article and approved the submitted version.

## Funding

This study was supported jointly by special funds for Taishan Scholars Project (grant no. tsqn201812149) and Academic Promotion Programme of Shandong First Medical University (2019RC004).

## Conflict of Interest

The authors declare that the research was conducted in the absence of any commercial or financial relationships that could be construed as a potential conflict of interest.

## Supplementary Material

Supplementary Figure 1 The OS analysis for patients receiving docetaxel. **(A)** The OS analysis for patients receiving docetaxel from OAK study (*P *< 0.0001). **(B)** The OS analysis for patients receiving docetaxel from POPLAR study. (*P *= 0.0063)

Supplementary Figure 2 The OS analysis for patients based on bTMB score and PDL1 expression from OAK study. **(A)** The OS difference based on different bTMB score (Cutoff=16) (*P* = 0.647). **(B)** The OS difference based on different PDL1 expression (Cutoff=1%) (*P* = 0.479). **(C)** The OS difference based on different PDL1 expression (Cutoff=50%) (*P* = 0.0058).
