# Supplementary figures and images for: A 5-Genomic Mutation Signature Can Predict the Survival for Patients With NSCLC Receiving Atezolizumab
Source: Front Immunol. 2021 Jun 23;12:606027. doi: 10.3389/fimmu.2021.606027 (PMC8261129; doi:10.3389/fimmu.2021.606027)

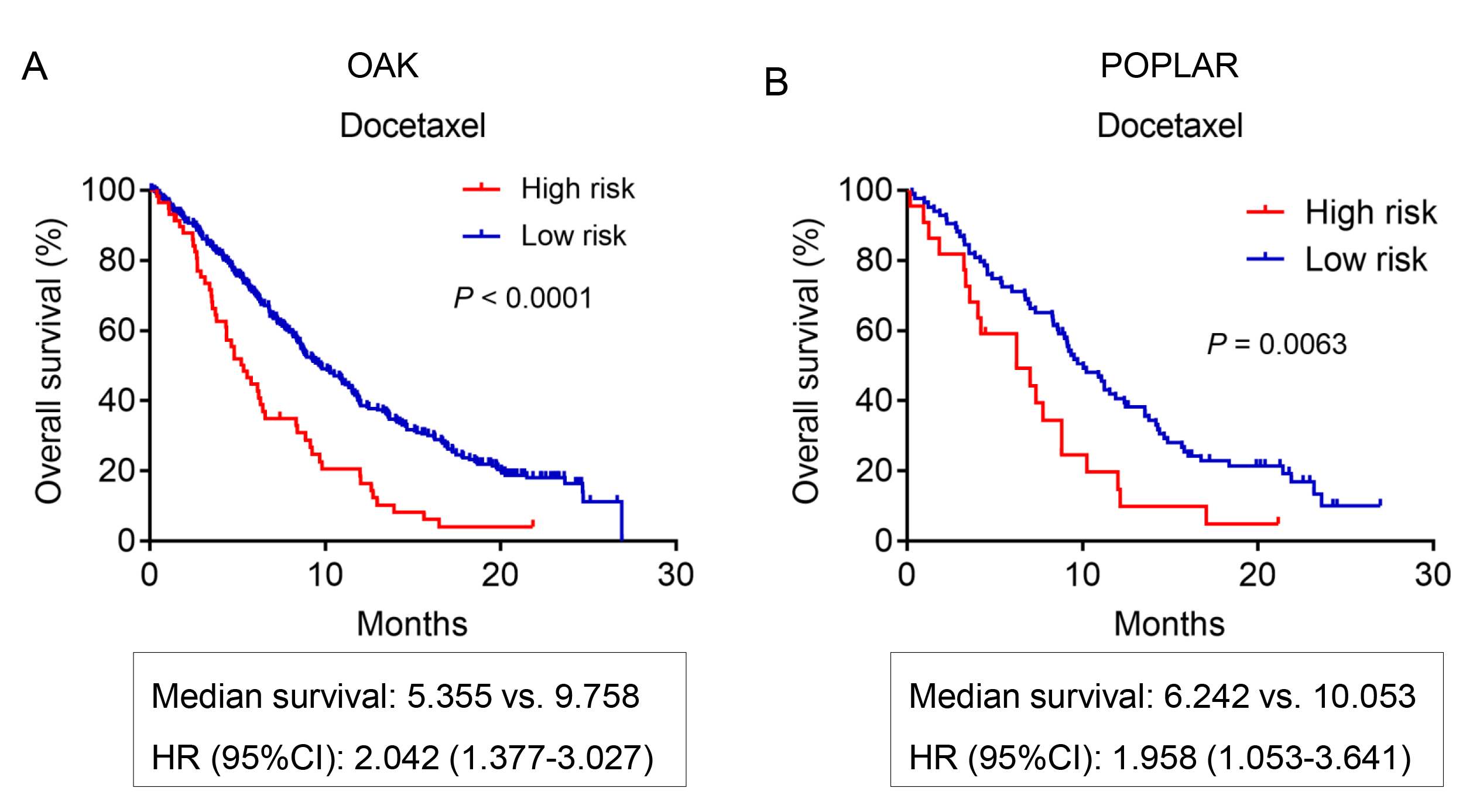

Supplement: Supplementary Figure 1 — The OS analysis for patients receiving docetaxel. (A) The OS analysis for patients receiving docetaxel from OAK study (P < 0.0001). (B) The OS analysis for patients receiving docetaxel from POPLAR study. (P = 0.0063) [file Image_1.tif]

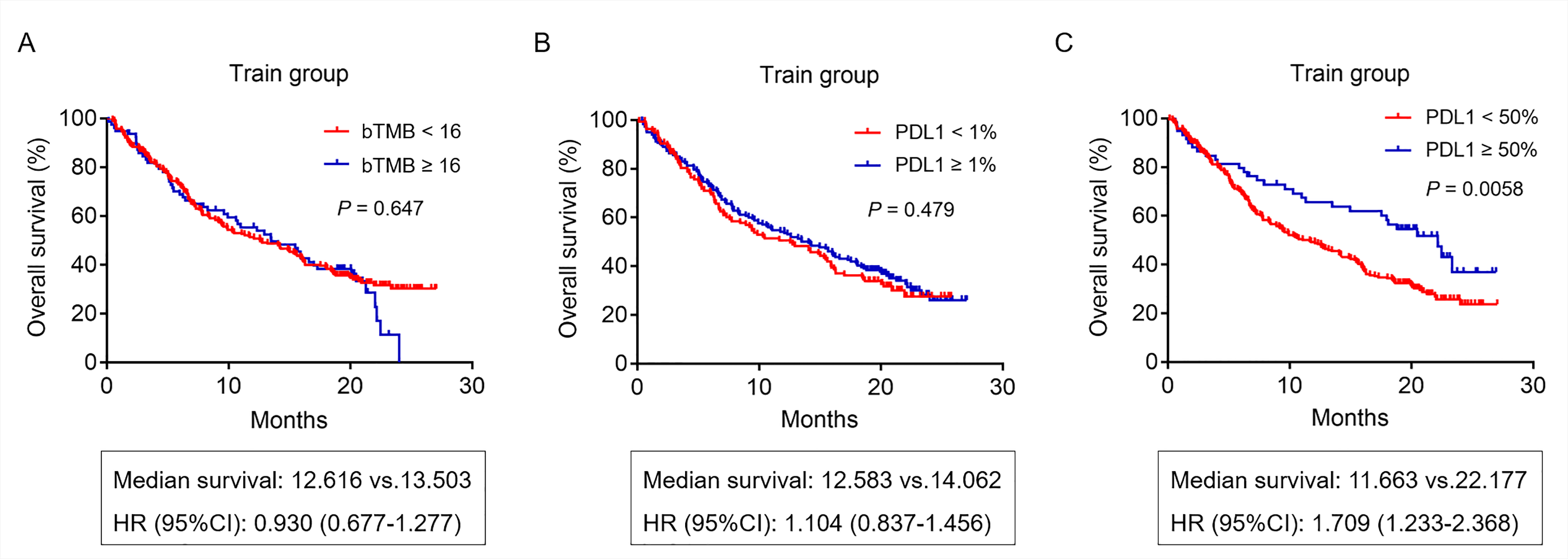

Supplement: Supplementary Figure 2 — The OS analysis for patients based on bTMB score and PDL1 expression from OAK study. (A) The OS difference based on different bTMB score (Cutoff=16) (P = 0.647). (B) The OS difference based on different PDL1 expression (Cutoff=1%) (P = 0.479). (C) The OS difference based on different PDL1 expression (Cutoff=50%) (P = 0.0058). [file Image_2.tif]
