# Supplementary material for: A 5-Genomic Mutation Signature Can Predict the Survival for Patients With NSCLC Receiving Atezolizumab
Source: Front Immunol. 2021 Jun 23;12:606027. doi: 10.3389/fimmu.2021.606027 (PMC8261129; doi:10.3389/fimmu.2021.606027)
Supplement: Supplementary file 3 [file DataSheet_1.doc]

Supplementary table 1. The HR and C-index based on different variables

| Variables | HR | C-index |
| --- | --- | --- |
| 5-gemomic mutation signature | 1.814 | 0.543 |
| bTMB | 0.930 | 0.498 |
| PDL1 (Cutoff=1%) | 1.104 | 0.513 |
| PDL1 (Cutoff=50%) | 1.709 | 0.538 |

Supplementary table 2: Univariate and multivariate Cox regression analysis of clinical variables affecting OS for patients receiving docetaxel based on training and testing group

| Variables | Univariate analysis | | Multivariate analysis | | Interaction* |
| --- | --- | --- | --- | --- | --- |
|  | Wald | *P* | HR (95% CI) | *P* | *P* |
| Age | 2.917 | 0.088 | NI |  | 0.416 |
| <65 |  |  |  |  |  |
| ≥65 |  |  |  |  |  |
| Race | 5.387 | 0.068 | NI |  | 0.207 |
| White |  |  |  |  |  |
| Asian |  |  |  |  |  |
| Others |  |  |  |  |  |
| Sex | 3.438 | 0.064 | NI |  | 0.381 |
| Female |  |  |  |  |  |
| Male |  |  |  |  |  |
| Histopathology | 7.036 | 0.008 |  | 0.018 | 0.398 |
| Squamous |  |  | Reference |  |  |
| Non-squamous |  |  | 0.760 (0.606-0.954) | 0.005 |  |
| ECOG | 21.254 | <0.0001 |  | <0.0001 | 0.162 |
| 0 |  |  | Reference |  |  |
| 1 |  |  | 1.666 (1.317-2.106) | <0.0001 |  |
| Smoking | 0.034 | 0.854 | NI | 0.180 | 0.837 |
| Current |  |  |  |  |  |
| Never |  |  |  |  |  |
| Previous |  |  |  |  |  |
| Risk model | 28.054 | <0.0001 |  | <0.0001 |  |
| Low risk |  |  | Reference |  |  |
| High risk |  |  | 1.995 (1.528-2.604) | <0.0001 |  |

NI: not included; * Interaction between variables and risk model
